# Bilateral Radial Ulnar Synostosis and Vertebral Anomalies in a Child with a *De Novo* 16p13.3 Interstitial Deletion

**DOI:** 10.1155/2013/149085

**Published:** 2013-07-01

**Authors:** Allison Tam, Kit Shan Lee, Sansan Lee, William Burkhalter, Lucio U. Pascua, Thomas P. Slavin

**Affiliations:** ^1^University of Hawaii, John A. Burns School of Medicine, Honolulu, HI 96813, USA; ^2^Hawaii Community Genetics, Ala Moana Building, 1441 Kapiolani Blvd, Suite 1800, Honolulu, HI 96814, USA; ^3^Kapiolani Medical Specialists, Hawaii Community Genetics, Honolulu, HI 96814, USA; ^4^Department of Pediatrics, University of Hawaii, John A. Burns School of Medicine, Honolulu, HI 96826, USA; ^5^Department of Surgery, University of Hawaii, John A. Burns School of Medicine, Honolulu, HI 96813, USA; ^6^Kapiolani Medical Specialists, Kapiolani Orthopedic Associates, Honolulu, HI 96826, USA; ^7^Department of Family Medicine, University of Hawaii, John A. Burns School of Medicine, Honolulu, HI 96789, USA

## Abstract

We describe an 8-year-old boy with developmental delay, clinical bilateral radial ulnar synostosis, Klippel-Feil anomaly, and other vertebral deformities who was found to have a *de novo* deletion of 114.5kb at 16p13.3. The deletion contains five genes and three miRNAs. The genes are *E4F1, DNASE1L2, ECI1, RNPS1*, and *ABCA3*; miRNAs are MIR3677, MIR940, and MIR4717. The specific deletion has never been previously reported. We describe the phenotype of the boy and review the genes in the deleted region. None of the regulatory elements have any known linkage to skeletal formation and/or maintenance. We believe this deletion is causative given that it was *de novo* and that this patient cannot be easily explained as having any other specific recognizable pattern of human malformation.

## 1. Introduction

Many of the reports on chromosome 16p13.3 gene deletions are in relation to the Rubinstein-Taybi syndrome, alpha thalassemia mental retardation, polycystic kidney disease, and familial Mediterranean fever. Chromosome 16p13.3 gene deletions associated with the Rubinstein-Taybi syndrome are discussed in depth in articles by Wallerstein et al. and Hennekam et al., respectively [1, 2]. We report a child with developmental delay, bilateral radial ulnar synostosis, Klippel-Feil anomaly, and other vertebral deformities with a *de novo* interstitial deletion of 114.5 kb on chromosome 16p found using chromosomal microarray. The deleted region contains five genes and three miRNAs of unclear clinical significance. We are not aware of reports of gene deletions that overlap with the 114.5kb deletion described here. The purpose of this paper is to describe our patient's unique phenotype and discuss the possible genotype-phenotype correlations of the deleted region.

## 2. Clinical Report

Past medical history was significant for term birth with complication of preeclampsia of unknown etiology. He had no neonatal complications. His birth weight was 7 lbs 3 oz. Family history was unremarkable for skeletal disorders. Younger full sibling sister was healthy without issues. The patient had a history of developmental delay and was in an early intervention program in California. He walked around age 2, spoke at age 3, and had phrases around age 4. He also was not toilet trained until age 4. Neurodevelopmental assessment at the age of 8 years showed a Wechsler Intelligence Scale for Children Fourth Edition (WISC IV) verbal comprehension of 89, working memory of 80, perceptional reasoning of 96, processing speed of 83, and low average full scale IQ of 84. He had a history of two febrile seizures, one at age 2 and one at age 6. The patient had no history of hospitalizations or surgeries. 

**Figure 1:**
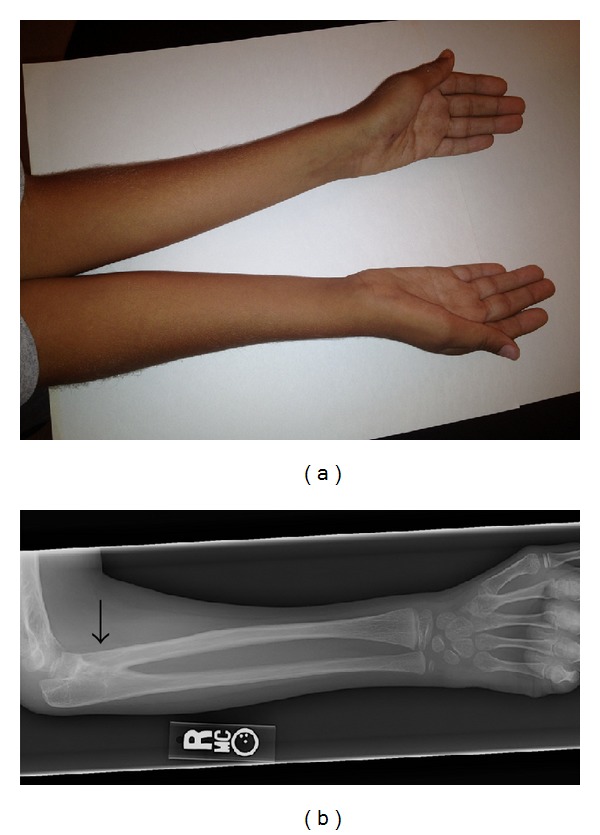
Photograph showing the patient's forearm with limitation of rotation (a). There is also hypoplasia of the thenar and hypothenar muscular prominences of his hands bilaterally. Radiograph of the right forearm (b). The arrow indicates the site of bony synostosis.

**Figure 2:**
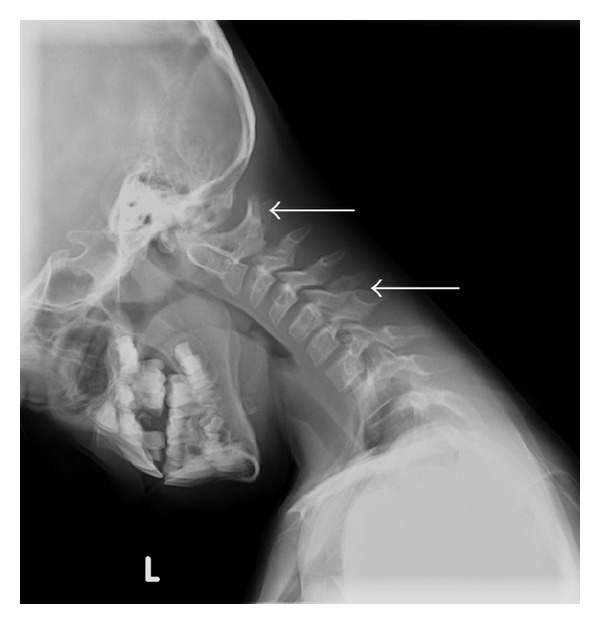
Radiograph depicting the Klippel-Feil anomaly with partial fusion of the posterior elements of C1-2 and C5-6 (arrows). Note also that there are 8 cervical vertebrae.

**Figure 3:**
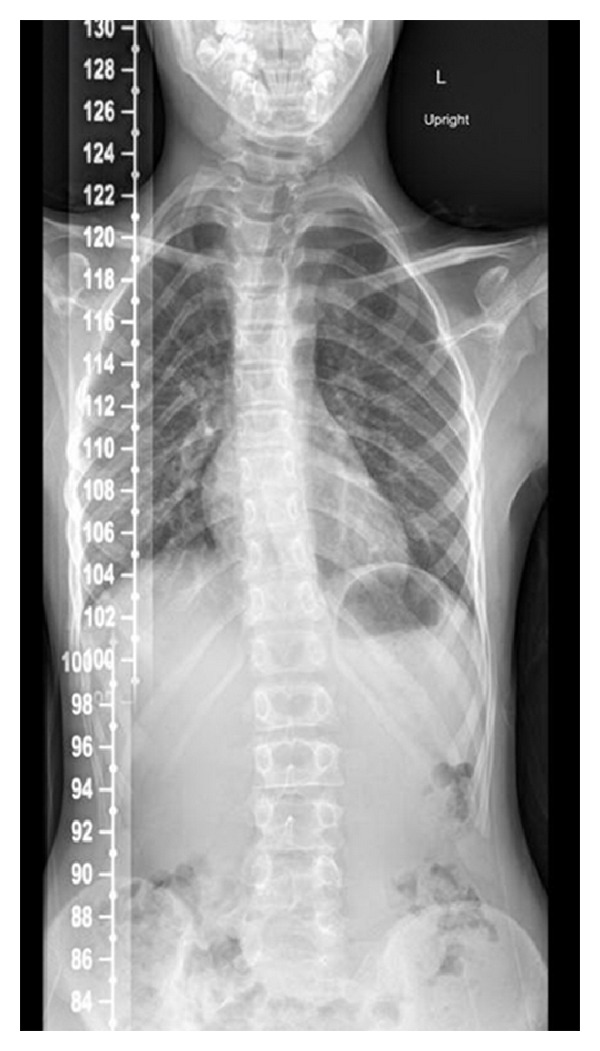
Chest radiograph from patient, showing 13 sets of ribs and “S” shaped scoliosis with 11-degree thoracic dextroconvex and 8-degree lumbar levoconvex scoliosis.

The patient's mother noted unusual arm positioning since birth. At the age of 7 the patient was referred to an orthopedist and was found to have bilateral radial ulnar synostosis by clinical exam (Figure 1(a)). Congenital fusions were also noted on cervical spine radiographs (Figure 2) obtained for restricted neck motion noted incidentally on physical examination. Plain radiographs of the right elbow revealed trochlear dysplasia and complete radial ulnar synostosis (Figure 1(a)). Dysplasia of the proximal radio-ulnar articulation was noted on the left leading to an incomplete, yet functionally nearly complete, synostosis. A true left bony synostosis was not confirmed on CT scan, although MRI was not completed to better define the fibrous ligament attachments. Radiographic assessment of the cervical spine was notable for eight cervical vertebrae and the Klippel-Feil anomaly with congenital fusions at C1-2 and C5-6. Imaging of the entire spine revealed other vertebral abnormalities including “S” shaped scoliosis with 11-degree thoracic dextroconvex and 8-degree lumbar levoconvex scoliosis, absence of the right pedicle at L5 with abnormal articulation with the sacrum, and 13 sets of ribs (Figure 3). 

At his initial genetics diagnostic evaluation at age 8, he measured 132.1 cm tall (75th percentile), weighed 23.12 kg (25th percentile), and his head circumference was 51.5 cm (20th percentile). He had nondysmorphic, but myopathic, facial features with a small jaw. His mouth displayed dental crowding with ankyloglossia, a large thick tongue, and a bifid uvula. He had prominent ear crus bilaterally. He had wide nipple spacing with the nipples being on the lateral aspect of his clavicles. He displayed limited range of motion in his neck, mild scoliosis, complete loss of forearm rotation on the right and limited pronation and supination on the left. He had a right transverse single palmar crease. He displayed hypoplasia of thenar and hypothenar musculature bilaterally (Figure 1). He had some prominence of the medial aspect to the top of his knees. His gait was uncoordinated and he was unable to run. The remainder of his physical exam was unremarkable. A renal ultrasound was normal. Full skeletal survey and echocardiogram were recommended but not done at the time of this report.

## 3. Cytogenetics and Molecular Studies

**Figure 4:**
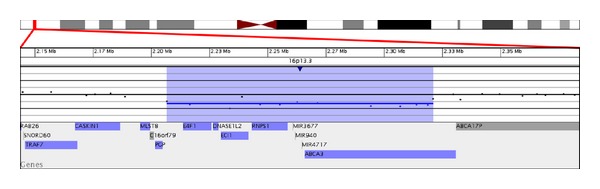
Microarray characterization of the 16p13.3 deletion described herein. Microarray plot showing single-copy loss of 13 oligonucleotide probes from 16p13.3, approximately 114 kb in size (chr16:2,206,663-2,321,155, hg18 coordinates). Probes are ordered on the *x*-axis according to physical mapping positions, with the most distal 16p13.3 probes to the left and the most proximal 16p13.3 probes to the right. Values along the *y*-axis represent log_2_ ratios of patient: control signal intensities. Results are visualized using Genoglyphix (Signature Genomics, Spokane, WA, USA).

Signature Genomic Laboratories, SignatureChiOS(Tm), version 2, 135 K microarray (Spokane, WA, USA) completed on blood showed a 114.5 Kb interstitial deletion, arr 16p13.3(2,206,663–2,321,155) × 1 dn (based on UCSC hg18 assembly) (Figure 4). Fluorescence in situ hybridization (FISH) analysis of interphase nuclei using a BAC clone from the deleted region confirmed a deletion, ish del(16)p13.3p13.3)(RP11-657D15-) dn. Parental analyses were performed and neither parent was found to carry a deletion or rearrangement of the 16p13.3 region. Thus the deletion identified in this patient was apparently *de novo* in origin. The deletion contains five genes and three miRNAs. The genes are *E4F1*, *DNASE1L2*, *ECI1*, *RNPS1*, and *ABCA3*; miRNAs are MIR3677, MIR940, and MIR4717 (Table 1). Complete blood count was normal and Fanconi's anemia testing by chromosome breakage was normal.

## 4. Discussion

A recent case report by Nelson et al. described a boy with a *de novo* 16p13.3 deletion with multiple congenital anomalies including tracheobronchomalacia, CT-proven metopic craniosynostosis, glandular hypospadias with severe ventral chordee, torticollis, esotropia, strabismus, fifth finger clinodactyly, hallux valgus, and global developmental delay [10]. He was found to have a *de novo* 555 Kb deletion at 16p13.3 deletion (chr16:2,716.773-3,271,348), which encompassed 25 known genes. This deletion does not overlap with the deleted segment described in this paper. Therefore, no clear link can be made between the bony abnormalities in the patient presented herein and the metopic craniosynostosis described by Nelson et al. [10]. 

Interestingly, none of the deleted elements have any known involvement with skeletal regulation to explain the striking bony defects seen in the patient presented herein. *E4F1, DNASEIL2, * and *RNPS1* are expressed in bone marrow and hematologic cell lines and have broad functions and therefore may have a role in bone regulation. *ECI1*, involved in beta-oxidation of unsaturated fatty acids, could be contributing to the patient's mild cognitive impairment; however, this seems unlikely given the absence of developmental regression or metabolic crises. *ABCA3*, involved in surfactant production, is the only gene in this region with a reported phenotype (Table 1). *ECI1* and *ABCA3* would only be expected to cause disease in an autosomal recessive fashion, likely explaining the lack of any history of neonatal respiratory distress syndrome and/or metabolic disease.

 Nonetheless, even though we cannot find a specific genotype-phenotype correlation to explain the bony findings in this patient, our findings are suggestive that this deletion is causative. The deletion was *de novo* and the patient does not fit any other syndromic phenotype besides Fanconi's anemia, which we completed testing for. We are not clinically working this child up for any other suspected condition. A sporadic birth defect could be a possible explanation; however, it would be unusual to involve only separate elements of the skeletal system. The deletion could be inducing positional effects in the region, thus affecting other genes in the surrounding area. Also, the miRNAs could be involved in bone remolding, or other bone regulatory abnormalities may have been unmasked from previously unknown recessive effects from the haploinsufficiency in the region. As this is the first case report involving this particular gene deletion, additional case reports and functional studies regarding the genes and regulatory elements contained in this region will be valuable in better understanding their role in skeletal regulation.

## Figures and Tables

**Table 1:** Deleted elements in the region with associated known function.

Deleted elements	Function
*E4F1 *	E4F transcription factor 1 is involved in the regulation of cell growth, survival, and/or differentiation [3]. Knockout mice studies suggest that E4F1 is crucial during early embryonic cell cycles and functions in mitosis [4]. Relatively high expression in peripheral blood-CD4+ T cells, heart, lungs, liver, and thymus [5]

*DNASEIL2 *	Deoxyribonuclease I- like 2, has strong endonuclease activity in the presence of certain cations, including CA2+ and Mg2+ [6]. Relatively high expression in pancreatic islets, liver, and bone marrow [5]

*ECI1 *	Dodecenoyl-coenzyme A delta-isomerase encodes an isomerase that is involved in the mitochondrial beta-oxidation of unsaturated fatty acids [7]. It has relatively high expression in heart, lungs, liver, kidneys, thymus, and whole brain [5]

*RNPS1 *	RNA-binding protein S1 is a serine-rich RNA binding protein involved in alternative splicing and regulation of physiological and developmental gene expression. It has been found in spliceosomes that trigger nonsense-mediated decay, which is thought to be involved in mRNA surveillance. RNPS1 is mainly expressed in hematologic cell lines [5]

*ABCA3 *	An ATP-binding cassette family member that is homologous to the studied *Caenorhabditis* elegans protein ced7. Involved in programmed cell death and expressed during embryogenesis. ABCA3 is highly expressed in lung, brain, pancreas, skeletal muscle, and heart and is known to play an important role in the formation of surfactant [5]. Homozygous mutations in ABCA3 have been shown in a cohort of patient with severe neonatal respiratory distress and surfactant deficiency [8]

MIR3677	A short novel noncoding RNA involved in posttranscriptional regulation. Little known function [5]

MIR4717	A short novel noncoding RNA involved in posttranscriptional regulation. Little known function [5]

MIR940	A short novel noncoding RNA involved in posttranscriptional regulation. Little known function. Reported expression in renal cell carcinoma [9]
